# Proton Density Fat Fraction Micro-MRI for Non-Invasive Quantification of Bone Marrow Aging and Radiation Effects in Mice

**DOI:** 10.3390/bioengineering12040349

**Published:** 2025-03-28

**Authors:** Hemendra Ghimire, Malakeh Malekzadeh, Ji Eun Lim, Srideshikan Sargur Madabushi, Marco Andrea Zampini, Angela Camacho, Weidong Hu, Natalia Baran, Guy Storme, Monzr M. Al Malki, Susanta K. Hui

**Affiliations:** 1 Department of Radiation Oncology, City of Hope National Medical Center, Duarte, CA 91010, USA; heghimire@coh.org (H.G.); mmalekzadeh@coh.org (M.M.); jilim@coh.org (J.E.L.); smadabushi@coh.org (S.S.M.); 2 MR Solutions Ltd., Ashbourne House, Guildford GU3 1LR, UK; marco.zampini@mrsolutions.com (M.A.Z.); angela.camacho@mrsolutions.com (A.C.); 3 Bio-Imaging Lab, Department of Biomedical Sciences, University of Antwerp, 2016 Antwerp, Belgium; 4 Department of Immunology & Theranostics, City of Hope National Medical Center, Duarte, CA 91010, USA; whu@coh.org; 5 Department of Hematology and Central Hematology Laboratory, Inselspital, Bern University Hospital, University of Bern, 3010 Bern, Switzerland; natalia.baran@insel.ch; 6 Institute of Hematology and Transfusion, Department of Experimental Hematology, 02-776 Warsaw, Poland; 7 Department of Radiotherapy, Universitair Ziekenhuis (UZ) Brussels, 1090 Brussels, Belgium; guy.storme@telenet.be; 8 Department of Hematology and Hematopoietic Cell Transplantation, City of Hope National Medical Center, Duarte, CA 91010, USA; malmalki@coh.org

**Keywords:** bone marrow imaging, proton density fat fraction, hematological malignancies, MRI biomarkers, histological validation

## Abstract

Background: Bone marrow (BM) adipocytes play a critical role in the progression of both solid tumor metastases and expansion of hematological malignancies across a spectrum of ages, from pediatric to aging populations. Single-point biopsies remain the gold standard for monitoring BM diseases, including hematologic malignancies, but these are limited in capturing the full complexity of loco-regional and global BM microenvironments. Non-invasive imaging techniques such as Magnetic Resonance Imaging (MRI), Computed Tomography (CT), and Positron Emission Tomography (PET) could provide valuable alternatives for real-time evaluation in both preclinical translational and clinical studies. Methods: We developed a preclinical proton density fat fraction (PDFF) MRI technique for the quantitative assessment of BM composition, focusing on the fat fraction (FF) within mouse femurs. We validated this method using aging mice and young mice subjected to 10 Gy X-ray irradiation, compared to young control mice. Water–fat phantoms with varying fat percentages (0% to 100%) were used to optimize the imaging sequence, and immunohistochemical (IHC) staining with H&E validated equivalent adipose content in the femur BM region. Results: Significant differences in FF were observed across age groups (*p* = 0.001 for histology and *p* < 0.001 for PDFF) and between irradiated and control mice (*p* = 0.005 for histology and *p* = 0.002 for PDFF). A strong correlation (R^2^~0.84) between FF values from PDFF-MRI and histology validated the accuracy of the technique. Conclusions: These findings highlight PDFF-MRI’s potential as a non-invasive, real-time, in vivo biomarker for quantitatively assessing the BM fat fraction in preclinical studies, particularly in studies evaluating the effects of aging, disease progression, and cytotoxic cancer therapies, including chemotherapy and radiation.

## 1. Introduction

Bone marrow (BM) adipocytes create a specialized microenvironment pivotal to the progression of both solid tumor metastases (e.g., breast and prostate cancers) and hematological malignancies (e.g., leukemia and multiple myeloma) [1,2,3,4,5]. These adipocytes promote tumor growth and survival by supplying lipids and secreting adipokines, which modify the BM niche to favor malignancy. Aging-associated increases in marrow adiposity further amplify these effects, compounding disease progression [6]. However, current approaches like single-point biopsies are invasive and fail to capture the spatial heterogeneity and complexity of the BM microenvironment. These limitations hinder the ability to assess dynamic changes in marrow composition and function during disease progression or therapeutic intervention [6,7,8]. The development of non-invasive imaging techniques in preclinical mouse models is crucial for delivering high-resolution, as well as comprehensive, insights into the spatial and functional dynamics of BM. Such advancements would enable precise assessment of adipocyte–cancer interactions, disease progression, and responses to therapeutic interventions, in translational and clinical research.

Magnetic Resonance Imaging (MRI), along with technologies like Computed Tomography (CT) [9], dual-energy X-ray absorptiometry (DXA) [10], and optical imaging, has advanced the in vivo quantification of BM adipose composition [11,12,13]. Among these, MRI is particularly valuable due to its ability to provide high-resolution images without exposing patients to ionizing radiation. Proton density fat fraction (PDFF) MRI quantifies BM fat by distinguishing between fat and water signals using chemical shift encoding [14,15,16]. While clinical studies increasingly leverage PDFF-MRI for evaluating BM composition and heterogeneity, its feasibility and accuracy in preclinical studies are not fully explored. This is particularly challenging in mice, given their smaller BM compartments and lower fat content compared to humans [17], which demand higher spatial resolution for accurate imaging.

At higher magnetic field strengths, such as 7 Tesla (7 T), achieving accurate PDFF measurements requires advanced techniques. These include multiple gradient echoes with short echo-to-echo spacing to capture the relative in-phase and out-of-phase signals of water and fat. Similar to other MRI advancements like T2-MRI for assessing BM inflammation [18], dynamic contrast-enhanced MRI (DCE-MRI) for leukemia-induced changes [19], and multiparametric approaches for quantifying myeloproliferative neoplasms [20,21], PDFF-MRI in mice femur BM requires further validation. Establishing standardized protocols and correlating PDFF-MRI findings with histological data are critical to bridging preclinical and clinical applications.

This study introduces the development of PDFF-MRI for assessing BM adiposity in mouse femurs, using three distinct experimental groups: aging mice, young mice exposed to 10 Gy X-ray irradiation, and control groups. Mouse models offer valuable insights into BM adiposity [17], as studies have shown it increases with age [22] and is influenced by X-ray irradiation, which in turn affects marrow adipocyte repopulation [11]. The research follows a three-step process: (1) evaluating PDFF linearity using a water–fat phantom, (2) performing PDFF-MRI on live mice femurs, and (3) validating findings through histological quantification of local BM adiposity. A phantom study is essential to optimize imaging parameters and minimize potential errors in vivo [23]. Correlating PDFF-MRI results with histological data further ensures the technique’s reliability in preclinical research [17,24]. This validation approach is essential for optimizing imaging protocols; advancing hardware and translational methodologies; and improving our understanding of BM disease mechanisms and treatment responses in mouse models, paving the way for clinical application in humans.

## 2. Materials and Methods

This cross-sectional study investigated the feasibility and efficacy of using PDFF-MRI to monitor FF in mouse femurs. All procedures were performed in compliance with the Institutional Animal Care and Use Committee (IACUC) at City of Hope (COH) National Medical Center, Duarte, CA, USA. Mouse models representing aging, X-ray irradiation, and control groups were utilized for this research.

### 2.1. Phantom Preparation

Water–fat phantoms with varying fat percentages (0%, 5%, 10%, 20%, 40%, 60%, and 100% by volume) were prepared using a mixture of gelatin, deionized water, emulsifier washing liquid, and commercially available peanut oil. Peanut oil was selected for its nuclear magnetic resonance properties, which closely resemble triglycerides in adipose tissue [25]. To prepare the phantoms, a solution of 0.1596 g of 225 bloom gelatin (Specialty Food Source, Brockton, MA, USA) per milliliter of deionized water was autoclaved at 121 °C for 15 min, then cooled to 50–55 °C. The solution was maintained within this temperature range on a laboratory hot plate magnetic stirrer with a magnetic mixer [26,27]. Next, variable amounts of peanut oil were added, and the mixture was emulsified using the BV1000 Bench Mixer Vortex Mixer (Benchmark Scientific Inc., Sayreville, NJ, USA) (115 V) at a maximum speed of 3200 RPM. The mixing duration was selected depending on the fat concentration, ranging from 5 min for 5% fat to 60 min for 60% fat. Microbubbles were removed using an ultrasonic bath (Branson Ultrasonic Corporation CPXH, Danbury, CT, USA), and the final solution was imaged using a micro-CT scanner (Precision X-ray) with a pixel resolution of 0.1 × 0.1 mm^2^ to verify the absence of microbubbles.

### 2.2. Mouse Models of Aging and X-Ray Irradiation

For the aging study, 96-week-old C57BL/6J mice (JAX 000664, Jackson Laboratory, Bar Harbor, ME, USA) were used. These mice were originally acquired at 93 weeks of age and were housed in the COH animal facility for the next 3 weeks until the time of MRI imaging studies.

For the X-ray irradiation study, 9–12-week-old C57BL/6N mice (NCI-Charles River Laboratory) were divided into two groups: (1) Control group: 5 untreated mice; (2) Irradiated group: 5 mice subjected to 10 Gy X-ray irradiation to the right leg. Irradiation was performed using a 3D image-guided, focused X-ray irradiation system (X-RAD SmART Precision X-ray machine, Precision X-Ray, North Branford, CT, USA) operating at 225 kV and 13 mA.

### 2.3. Mouse Preparation and Monitoring for MRI Scanning

Mice were anesthetized using 2–4% isoflurane delivered at a flow rate of 2 L/min of oxygen throughout the imaging procedure. To maintain stable body temperature at 37 °C during the experiment, a Minerve multi-station temperature control unit (Esternay, France) was utilized.

Respiration rates were also kept stable at 40–50 breaths per minute and monitored using an MR-compatible small-animal monitoring and gating system (SA Instruments, Inc., Stony Brook, NY, USA), with a respiration pad secured to the mouse. Initially, a surface coil was employed over the femur region to enhance signal quality. However, this setup proved time-consuming and required rigorous management to prevent body temperature drops, especially during extended sessions. To address these challenges, a body coil with integrated temperature control was adopted. The coil supported better physiological stability, minimized stress, and reduced the risk of hypothermia. Mice were then positioned supine on standard bedding, with femurs aligned in the same plane using flat popsicle stick slabs, ensuring proper anatomical positioning and coplanarity for imaging.

### 2.4. Image Acquisition and Analysis

MRI data were acquired using a multi-echo gradient-echo sequence with the following parameters: 6 different echo times (TEs) = 3760, 4080, 4400, 4720, 5040, 5360 µs; time repetition (TR) = 180 ms; field of view (FOV) = 22.55 × 30.00 mm^2^; slice thickness = 1 mm; spacing between slices = 0.10 mm; matrix size = 128 × 128; flip angle = 25°; bandwidth = 66.7 KHz; number of excitations (NEX) = 2; pixel size = 0.1758 × 0.2344 mm^2^.

Water–fat decomposition was performed using the GOOSE (GlObally Optimal Surface Estimation) algorithm implemented in the MR Solutions scanner software (PreClinical Scan 4.1.1.08). This algorithm accurately separates fat and water signals for precise FF quantification, with an emphasis on reducing swaps artifacts often associated with fat–water decomposition challenges [28].

For FF analysis, region-specific masks were created on T2-weighted images using MATLAB R2024b. These masks were thoroughly co-registered to align with corresponding anatomical locations and orientations in both histological sections and PDFF-MRI images, enabling precise comparisons and analyses.

### 2.5. Tissue Preparation

Following MRI imaging, femurs were harvested from euthanized mice and processed for histological analysis. Freshly dissected femurs were fixed in 10% paraformaldehyde (PFA) at 4 °C overnight to preserve tissue components and morphology.

### 2.6. Hematoxylin and Eosin (H&E) Staining and Histological Analysis

After fixation, femurs were washed three times with phosphate-buffered saline (PBS) and decalcified in 10% ethylenediaminetetraacetic acid (EDTA) solution at room temperature for 48 h. Decalcified femurs were rinsed with PBS and embedded in paraffin to create tissue blocks. Sections were deparaffinized, rehydrated, and stained with Modified Mayer’s H&E Y Stain (America MasterTech Scientific, Lodi, CA, USA) using an H&E Auto Stainer (Prisma Plus Auto Stainer, Sakura, Japan), following standard laboratory protocols.

H&E-stained slides were scanned using a NanoZoomer S360 Digital Slide Scanner (Hamamatsu, Japan), and whole slide images were analyzed with NDP.view2 software. Subsequently, Fiji/ImageJ (1.53t) was employed to generate representative images, while MATLAB R2024b was used to quantify the adipocyte-occupied area regions defined by PDFF-MRI analysis.

Distal femurs were selected for validation due to their higher adipose content and easier handling, particularly in aged or irradiated mice with brittle bones, enabling precise sectioning and analysis.

### 2.7. Statistical Analysis

Statistical analyses were performed using GraphPad Prism ver. 8 (GraphPad Software Inc., La Jolla, CA, USA). Group comparisons were conducted using one-way ANOVA with Tukey’s post hoc test, with statistical significance set at *p* < 0.05. R^2^ values were calculated in Excel to assess correlations between histological and PDFF-MRI data, as well as results from phantom studies, using linear regression. Data are presented as mean ± SEM, with significance levels denoted as follows: ns = not significant, * *p* < 0.05, ** *p* < 0.01, *** *p* < 0.001.

## 3. Results

**Figure 1:**
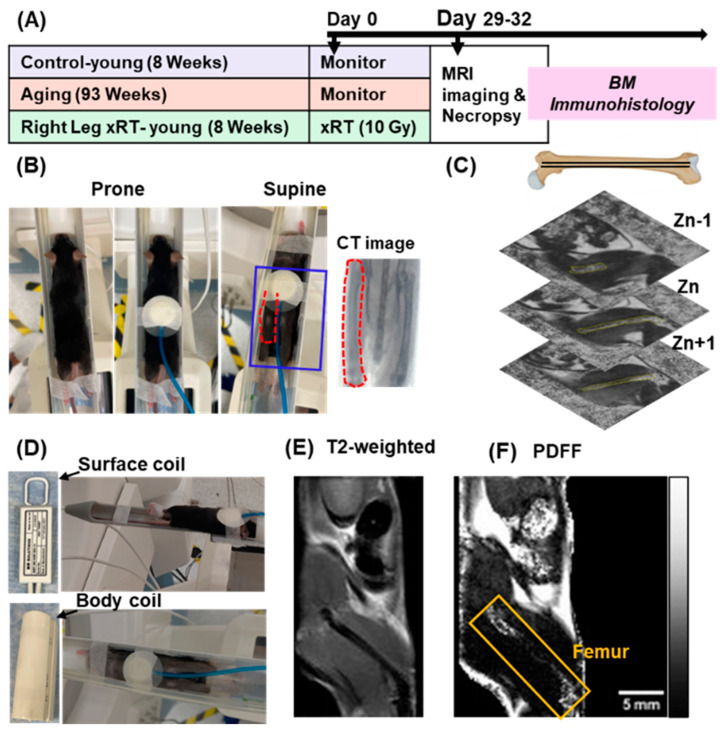
Technological development for MRI-based assessment of FF in mouse femur BM. (**A**) Schema of the experimental design, showing the handling of mouse and tissue samples over time. (**B**) The mouse is positioned in both prone and supine orientations for optimal alignment of femurs and marrow within the imaging plane. The supine positioning allows for the easier connection to ECG gating and precise temperature control. The accurate positioning of the femurs for the entire region of marrow imaging is further confirmed through CT scans, followed by MRI imaging. (**C**) Optimization of imaging parameters, including resolution, slice thickness, and inter-slice spacing, to have a clearer visualization of fat content within BM region. Z-stack slicing in the aligned femur was illustrated using a femur prepared in BioRender(version 4.0; https://BioRender.com/9yzrw68, (accessed on 24 March 2025)). (**D**) Comparative testing of custom-built surface (femur) coils and a conventional body coil for PDFF-MRI. The body coil was selected due to its similar sensitivity for PDFF-MRI imaging and the additional benefit of maintaining animal warmth. (E-F) Representative T2-weighted (**E**) and PDFF (**F**) images showing the right femur BM in sagittal orientation. Herein, representative T2-weighted and PDFF images were prepared using a 19-week-old healthy mouse.

The development of MRI techniques for quantifying FF in mouse femur BM is summarized in Figure 1. This approach enabled precise, artifact-free PDFF-MRI imaging, a critical advancement for preclinical translational studies investigating BM adipose content.

### 3.1. Experimental Design and Positioning

Outline of the experimental workflow, including mouse handling, imaging setup, and data acquisition, is shown in Figure 1A. Proper alignment of the femur’s central core was essential for accurately imaging BM adipose content. Comparative tests of prone and supine positioning (Figure 1B) demonstrated that supine positioning had superior femur alignment, facilitating complete BM visualization. Thus, all subsequent MRI studies were executed utilizing the supine positioning.

To minimize motion artifacts caused by respiration, an initial custom bedding platform was built for use with a surface coil (Figure 1D). While this initial setup contributed strongly to improved signal acquisition, it was still time-intensive and difficult to maintain field of view (FOV) adjustments. Thus, a standard bedding platform, optimized for use with a body coil, was used. This setup is easier for FOV adjustments, streamlining the imaging process, and to preserve image quality. Femur positioning and axial alignment were subsequently validated using CT scans followed by MRI, ensuring optimal alignment for precise BM imaging.

### 3.2. Optimization of MRI Parameters

Key MRI parameters, including resolution, slice thickness, and inter-slice spacing, were optimized to ensure clear imaging of the small adipose regions within the BM. Parameters were adjusted to ensure that at least one z-stack slice encompassed the entire marrow region from the distal to proximal femur (Figure 1C). The FOV was carefully focused on the lower portion of the mouse’s body, particularly the right femur, to ensure comprehensive coverage of the BM region. Representative sagittal images from T2-weighted (Figure 1E) and PDFF (Figure 1F) scans demonstrate the effectiveness of the designed imaging protocol in producing high-resolution, artifact-free PDFF images suitable for subsequent analyses.

### 3.3. Validation of PDFF Measurements Using Water–Fat Phantom Models

**Figure 2:**
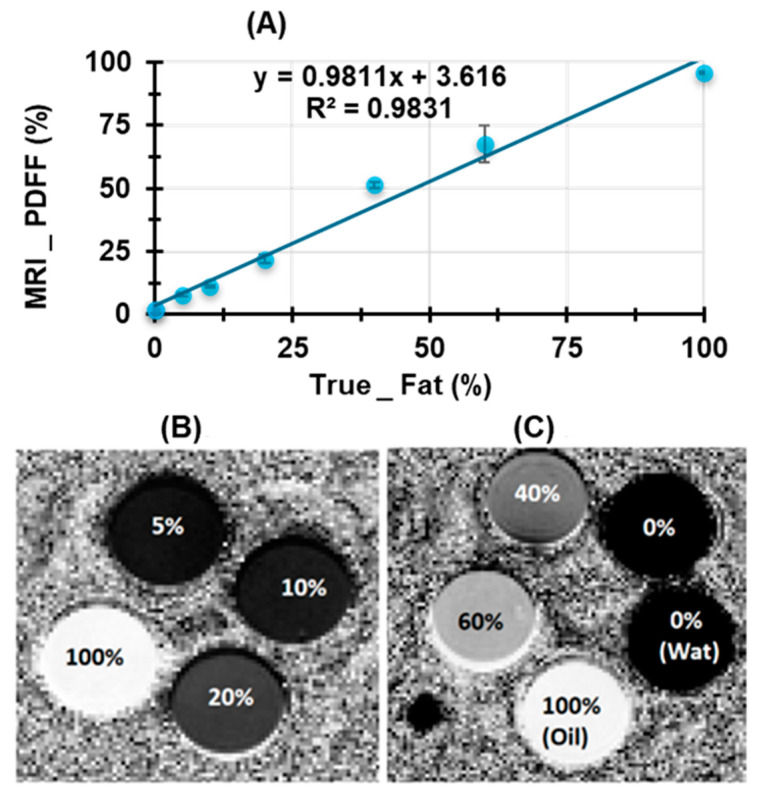
Water–fat phantom study showing strong correlation between measured and true FF. (**A**) Correlation between true fat volume fraction and the measured mean FF using MRI. (**B**) FF maps display fractions of 5%, 10%, 20%, and 100% fats. (**C**) FF maps indicating 0% (water), 0% (Gelatin and Water), 40%, 60%, and 100% (oil).

The water–fat phantom studies revealed a strong correlation between the known fat fraction and the measured PDFF values obtained using a 7 T preclinical MRI system, with an R^2^ value of 0.9831 (Figure 2A). A slight overestimation was observed in the pure water sample, which was in line with findings reported in the literature [29], indicating that such discrepancies are common in fat quantification techniques at high magnetic field strengths.

FF maps for fat concentrations of 5%, 10%, 20%, and 100% effectively demonstrated the system’s ability to differentiate between varying fat compositions (Figure 2B). Furthermore, Figure 2C highlights the imaging response to samples with different compositions: 0% (water), 0% (gelatin and water), 40%, 60%, and 100% (oil). The standard deviation observed in the 60% fat sample likely results from microbubbles (<100 μm), which might have influenced the measured values. These findings confirm the reliability and accuracy of the 7 T preclinical MRI system for assessing fat fractions in phantom models.

### 3.4. Effects of Irradiation and Aging on Bone Marrow Fat Fraction

**Figure 3:**
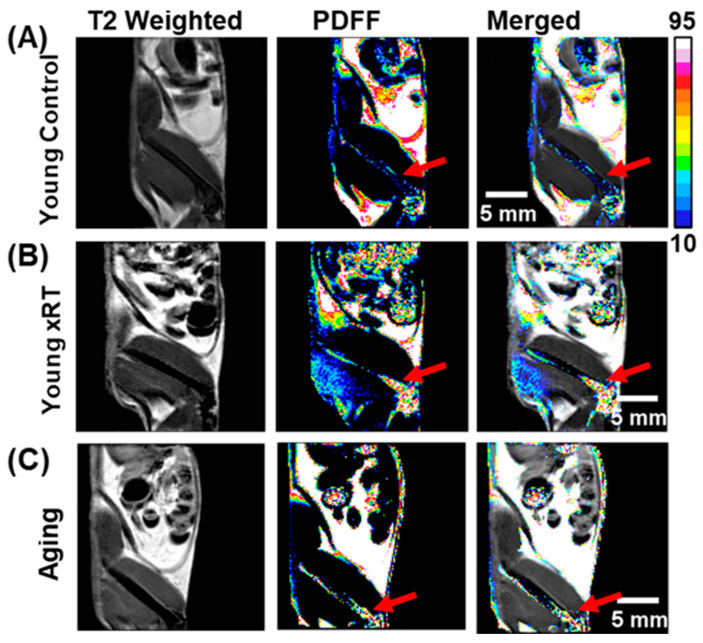
T2-weighted and PDFF-MRI sequences of the mouse body, focusing on the BM region in the right femur. (**A**) Representative images from a young control mouse, showing a mid-plane view of the right femur in T2-weighted, PDFF, and merged images. (**B**) Images of an irradiated mouse model, showing irradiation-associated changes in FF within the corresponding femur BM. (**C**) Images from an aged mouse, demonstrating age-related changes in fat distribution. The PDFF and merged images provide enhanced visualization of fat distribution within the body and femur BM due to both irradiation and age. The red arrows point to the distal femur marrow, where FF levels are consistently higher compared to mid-shaft, and proximal areas in both irradiated and aged mice.

We evaluate the impact of irradiation and aging on BM composition, particularly FF, using T2-weighted and PDFF-MRI sequences. These imaging modalities were employed to investigate FF changes in the BM of the right femur in different mouse models. Figure 3 depicts T2-weighted, PDFF, and merged PDFF/T2 MRI sequences, providing a detailed visualization of the FF distribution within the femur BM and surrounding tissues. Representative images from healthy young control mice showed low FF levels in the BM, indicative of a well-maintained hematopoietic environment (Figure 3A). These baseline images served as a reference for comparisons with irradiated and aged mice. In mice subjected to X-ray irradiation at the dose of 10 Gy, PDFF maps demonstrated a striking increase in FF, particularly in the distal femur region (Figure 3B). This rise in adiposity reflects irradiation-induced disruption of the BM cellular microenvironment with fat accumulation associated with impaired hematopoiesis and diminished regenerative capacity. Such changes might suggest a transition toward a less supportive BM niche, with potential serious implications for recovery and immune function following irradiation [30]. Similarly, in aged mice, BM composition displayed a pronounced increase in FF, indicative for a rise in fat content, (Figure 3C). Age-related fat accumulation within BM changes its microenvironment, diminishing its hematopoietic potential and contributing to age-associated bone conditions, such as osteopenia with decreased bone density, or osteoporosis with increased fragility [22].

### 3.5. Correlation of Histological and MRI-Based Bone Marrow Fat Fraction Analysis

**Figure 4:**
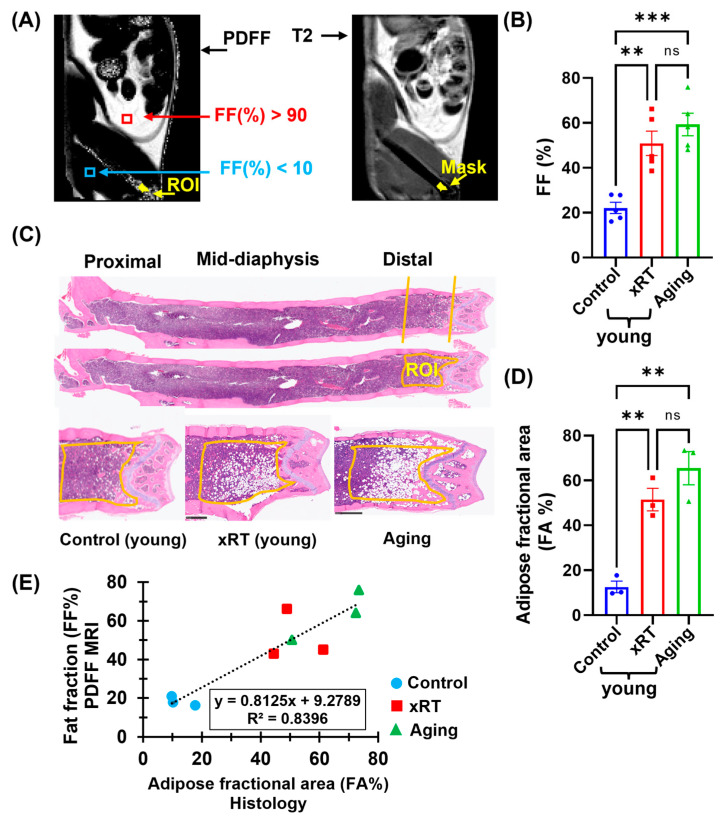
Quantification of FF (%) in distal femur BM of studied mouse groups. (**A**) Representative PDFF image of the right femur side of an aging mouse. Abdominal fat exhibits higher than 90% FF (highlighted by the red-lined square), while femur muscle (blue square) is less than 12%. A mask was created using a T2-weighted image, focusing on a 1.5–2 mm region within the marrow, starting 1 mm away from the distal femur (lateral condyle). (**B**) Quantitative FF (%) in the region of interest (ROI) of young control (Control), young irradiated (xRT), and aging mice using PDFF-MRI images. FF (%) significantly increases in the ROI of irradiated and aging mice compared to control mice (*p* < 0.001). (**C**) H&E-stained femur BM sections (proximal, mid-diaphysis, and distal parts) from a control mouse. The H&E-stained distal section (yellow-lined region) represents a 1.5–2 mm area near the popliteal surface, adjacent to the synovial membrane reflection line, which corresponds to the same region used for FF quantification in PDFF-MRI imaging. Comparisons were made between young control, 10 Gy X-ray irradiated, and aging mice. (**D**) Quantification of the adipose fractional area in the distal femur BM (highlighted in (**C**)) showing significantly increased adiposity in irradiated and aging mice compared to controls (*p* < 0.01). Three mice (*n* = 3) were used in each group. (**E**) Correlation between the adipose fractional area from H&E-stained images and the integrated density from PDFF-MRI images. Data points for control mice are shown as blue circles, irradiated mice as red squares, and aging mice as green triangles. A strong correlation is observed, with an R^2^ value of 0.84, indicating consistency between histological and MRI-based fat quantification. Data are presented as mean ± SEM, with significance levels denoted as follows: ns = not significant, ** *p* < 0.01, *** *p* < 0.001.

Next, we focused on validating PDFF-MRI as a reliable tool for assessing BM adiposity by comparing it with histological analyses of H&E-stained femur sections. We aimed to establish the anatomical variability of fat content, to quantify BM adiposity across different mouse groups, and ultimately to correlate MRI findings with histological data. Figure 4 illustrates the relationship between the PDFF-MRI and histological findings. A representative PDFF image of an aging mouse demonstrates distinct fat content variations (Figure 4A). Abdominal fat regions (red square) exhibit ~90% FF, while femoral muscle regions (blue square) display < 12% FF. These findings highlight the anatomical variability of fat distribution. Furthermore, mean FF values from the BM regions of each group were derived from regions of interest (ROIs), identified in T2-weighted images (Figure 4B). Aging mice exhibited the highest FF in femur BM, indicating a significant age-related increase in adipocyte accumulation. Similarly, irradiated mice displayed elevated FF compared to controls (*p* < 0.0013). Increased BM adiposity, particularly in aged and irradiated models, aligns with evidence linking fat accumulation to impaired hematopoiesis and diminished marrow resilience.

H&E staining of histological sections of mouse femurs was further evaluated for BM adiposity calculation. H&E-stained sections of the distal femur, selected for its ease of sectioning and high adipocyte density, further confirmed BM adiposity. Representative histological images highlight a significant increase in the adipocyte population (Figure 4C) within the BM of aging and irradiated mice, compared to controls. Magnified black-and-white sections illustrate the adipose fractional area (FA) in these groups, supporting the MRI findings (PDFF images). For statistical comparison, we then conducted quantification of FA, which revealed significantly higher adiposity in aging mice and irradiated femurs (*p* < 0.01) compared to controls, corroborating the MRI results (Figure 4D). Finally, following the comparison of MRI results with histology, a strong correlation (R^2^ = 0.84) was observed between FA from histological sections and FF from PDFF-MRIs, as shown in Figure 4E. This high correlation confirms the consistency between histology and MRI-based measurements, demonstrating the reliability of PDFF-MRI for preclinical BM adiposity assessment.

Taken together, these results support the use of PDFF-MRI as a robust, non-invasive technique for studying BM adiposity and its implications in preclinical models of aging- and irradiation-induced BM changes.

## 4. Discussion

This study demonstrates the feasibility of using preclinical MRI to assess PDFF within the femoral marrow by monitoring local FF in aging, young, and irradiated young-mouse models. A critical component of this validation involved the use of a water–fat phantom to optimize imaging parameters, including the resolution and number of averages, for the GOOSE algorithm. The phantom study provided a controlled environment to refine imaging settings, ensuring the accuracy and reliability of PDFF measurements while minimizing errors in transitioning to in vivo applications [23].

The technical feasibility of PDFF-MRI for femoral BM was further validated through comparisons with histological analyses. The PDFF-MRI measurements of marrow FF showed a strong correlation with adipose volumes observed in histological sections of local regions (R^2^~0.84). This strong agreement underscores the potential of PDFF-MRI to deliver precise and real-time insights into BM composition, particularly its adipose tissue content, thus offering a powerful tool for understanding BM pathophysiology.

Additionally, the study highlights the importance of selecting appropriate preclinical mouse models for capturing biological variability in BM adiposity. In contrast to synthetic phantom studies, animal models provide the biological context necessary to study disease-specific changes in BM architecture [31]. Therefore, the present study includes phantom models complemented by diverse preclinical mouse models relevant to oncology and aging, enabling the capture of adipose variability in the BM region and providing a solid foundation for further translational research.

The present preclinical study has several clinical implications for understanding BM microenvironment alteration across various diseases, as outlined in Table 1. The heterogenous nature of the BM microenvironment, especially BM adipocytes, is increasingly recognized as critical to evaluate the adverse effect of treatment on bone marrow and the sanctuary for disease progression [32]. This has drawn interest in conditions such as hematologic malignancies, osteoporosis, sickle cell disease (SCD), and marrow failure syndromes, to name a few [33]. Our previous studies showed that leukemia progression and residual disease influence BM niche components, including local FF [4], and that BM composition undergoes substantial changes with age [11,12,13]. These findings suggest that temporal and spatial variations in marrow FF could serve as potential imaging biomarkers for BM-related health issues, especially those affecting bone density, immune function, and metabolic health. PDFF-MRI could be particularly valuable for monitoring the effects of cancer treatment on the marrow fat fraction (FF) as a marker of treatment-related adverse effects on bone and BM in gynecologic and breast cancer patients [11,30]. However, FF alterations due to conditioning regimens (chemotherapy and radiation) before hematopoietic stem cell transplantation (HCT) or bone marrow transplantation (BMT) require thorough translational validation, as donor marrow supports the repair and regeneration of conditioning-induced damage. For example, advancements in radiation modalities such as total marrow irradiation (TMI) [34] and total marrow and total lymphoid irradiation (TMLI) [35] are being evaluated for their potential to control disease within BM through dose escalation to the entire skeletal system while minimizing radiation exposure to vital organs like the lung, liver, and gut [36]. However, little is known about their impact on marrow regeneration, warranting further investigation. Preclinical validation of these approaches is vital to understand their impact on BM regeneration and bone health, prevent BM damage, or develop precision treatments that mitigate these damages [30,37,38,39].

Reverse translational efforts from clinical to preclinical settings remain necessary to gain deeper insights into the complexities of BM treatments and diseases [31]. Developing reliable PDFF-MRI techniques for mouse models is critical for capturing both regional and global complexities of BM, including adipocyte distribution, hematopoietic cellularity, and overall tissue function. These non-invasive approaches like PDFF-MRI overcome the limitations of traditional biopsies, enabling earlier detection, improved disease monitoring, and more effective therapeutic interventions. Our developed technique allows for simultaneous adipose assessment of femurs and muscle fat within a single scan, making it suitable for longitudinal studies.

Despite the promising results of this study, it has some limitations. First, while PDFF-MRI provides a non-invasive method for assessing the BM fat fraction, its spatial resolution is lower than that of histological techniques, making it harder to capture fine molecular- or cellular-level changes. Future studies should focus on optimizing MRI acquisition parameters and incorporating high-resolution imaging techniques to improve spatial precision. Higher-resolution PDFF-MRI could enable a more detailed assessment of the spatial heterogeneity of adipocyte distribution within the femur, which may be biologically relevant for evaluating the BM niche composition and function. Additionally, integrating advanced deep learning algorithms for image processing could improve fat–water decomposition accuracy and minimize artifacts. Second, this study primarily employed a cross-sectional design, limiting our ability to track dynamic changes in BM adiposity over time. Longitudinal studies with repeated imaging sessions would provide deeper insights into marrow composition changes with aging, disease progression, and therapeutic interventions. Finally, while our study demonstrated a strong correlation between PDFF-MRI and histological adiposity measurements, further validation across diverse preclinical models and clinical datasets is necessary to establish PDFF-MRI as a standardized imaging biomarker for BM assessment. To bridge preclinical findings with clinical applications, we are currently conducting a clinical correlative study (NCT03422731) measuring longitudinal changes in BM percentage FF using PDFF-MRI in allogeneic transplant patients. Correlating imaging findings with pathological data will provide critical insights into the translational relevance of PDFF-MRI for clinical BM assessment.

## 5. Conclusions

In conclusion, this study established PDFF-MRI as a reliable and non-invasive tool for assessing BM fat content, paving the way for its application in understanding BM dynamics in various pathological conditions. The development and validation of PDFF-MRI in a preclinical setting is a major step forward in the non-invasive assessment of BM composition. By providing real-time, quantitative insights into BM fat, PDFF-MRI has the potential to transform how we study BM diseases in translational research and improve clinical outcomes. Future research should focus on refining this imaging technique, exploring its longitudinal applications, and validating findings in parallel with human studies.

## Figures and Tables

**Table 1:** Potential clinical applications of PDFF-MRI for BM assessment in mouse models.

Clinical Area	Potential Implication of PDFF-MRI
SCD and Marrow Failure Syndromes	To characterize BM microenvironment remodeling in SCD and marrow failure syndromes, supporting individualized treatment strategies and improving BM response monitoring to allogeneic BMT [33].
Hematological and Skeletal Metastases	To enable non-invasive monitoring of BM metabolic remodeling in leukemia and bone metastases, providing insights into disease progression and therapeutic response [4].
Pelvic Radiation and Chemotherapy for Gynecological Cancers	To evaluate BM damage from pelvic irradiation and chemotherapy, facilitating the development of BM-sparing radiation strategies to reduce hematologic toxicity [11,30].
Metabolic Disorders	To investigate BM fat as a potential biomarker for obesity and diabetes and its influence on hematopoiesis and immune function, potentially leading to novel therapeutic interventions [4].
BMT Conditioning Regimens	To assess the impact of advanced BM transplant conditioning strategies, such as TMI and TMLI, on local marrow damage, marrow regeneration, and graft failure, aiding in transplant optimization [34,35,36].
Osteoporosis and Aging	To quantify BM fat fraction to assess its relationship with bone density and fracture risk, supporting early diagnosis and intervention strategies [11,12,13].

## Data Availability

The datasets used and/or analyzed in this study are available from the corresponding author upon reasonable request.
